# Corrosion Behavior of Candidate Functional Materials for Molten Salts Reactors in LiF–NaF–KF Containing Actinide Fluoride Imitators

**DOI:** 10.3390/ma15030761

**Published:** 2022-01-20

**Authors:** Eduard Karfidov, Evgueniya Nikitina, Maxim Erzhenkov, Konstantin Seliverstov, Pavel Chernenky, Albert Mullabaev, Vladimir Tsvetov, Peter Mushnikov, Kirill Karimov, Natalia Molchanova, Alexandra Kuznetsova

**Affiliations:** The Institute of High Temperature Electrochemistry of the Ural Branch of the Russian Academy of Sciences, St. Akademicheskaya 20, 620066 Ekatherinburg, Russia; karfused@mail.ru (E.K.); m.erzhenkov@ihte.uran.ru (M.E.); gluk222@yandex.ru (K.S.); suzaku112@gmail.com (P.C.); albert_06@mail.ru (A.M.); vvc1@land.ru (V.T.); p.mushnikov@gmail.com (P.M.); Karimov.Kirill@gmail.com (K.K.); molchanova@ihte.uran.ru (N.M.); top.fairy.star@mail.ru (A.K.)

**Keywords:** corrosion, candidate materials, molten salts reactors, degradation, nickel, chromium, molybdenum, fluoride melts

## Abstract

Molten fluorides of alkali metals are considered a technological medium for molten salt reactors (MSRs). However, these media are known to be extremely corrosive. The successful implementation of high-temperature technological devices using molten alkali metal fluorides requires the selection of such structural materials that have high corrosion resistance in melts with compositional characteristic of MSRs. In this research, the corrosion behavior of 12Cr18Ni10Ti steel, the alloy Ni60Cr20Mo15, and the alloy Monel 404 (Ni50Cu50) was investigated in the LiF–NaF–KF eutectic melt, containing additions of CeF_3_ and NdF_3_ from 0 to 5 wt.% as imitator fluorides of actinides in an inert argon atmosphere at 550 °C for 100 h. Gravimetry, energy-dispersive X-ray (EDX) microanalysis of surfaces and cross-section of samples, and ICP-MS were used to establish the corrosion behavior of the investigated alloys. Corrosion resistance of the studied materials was found to decrease in a row from Monel 404 > Hastelloy C2000 > 12Cr18Ni10Ti. The addition of cerium fluoride into the melt resulted in the additional etching of the alloy surface. The addition of neodymium fluoride resulted in the formation of the point/inter-crystalline corrosion damages in the sample bulk. The samples of steel 12Cr18Ni10Ti were subjected to local cracking corrosion. The austenitic nickel-based alloys suffered specific local corrosion with formation of subsurface voids. Excellent corrosion resistance of the Monel alloy under the test conditions was found.

## 1. Introduction

Molten alkali metal halogenides are promising coolants, allowing for the expansion of the operating temperature range of power plants by hundreds of degrees, including nuclear reactors, to ensure efficient heat transfer from the hot zones of high-temperature devices.

Due to the thermal and radiation resistance in the temperature range from the melting point to the boiling point, halogenides compare favorably with water and organic heat carriers. In emergency situations, for example, in the case of damage or destruction to the pipeline or to the housing of the heat transfer device, they do not form explosive gas mixtures when interacting with water or a humid atmosphere. These properties, as well as basic thermophysical characteristics, make molten fluorides and chlorides of alkali metals attractive and competitive coolants, components of fuel mixtures, and regenerative media of new-generation nuclear reactors. The design of many high-temperature technological processes using molten salts, including newly developed ones, is associated with significant material science difficulties.

The significant aggressive action of alkali metal halide melts requires selection of resistant materials, development of methods of corrosion protection in molten salts, a detailed study of corrosion behavior, and the mechanism of destruction of materials.

Properties of molten fluoride salts enable their usage in molten salt reactors (MSRs) as solvents [1,2,3]. However, the successful usage of molten alkali fluorides [4,5,6,7] requires the development of functional materials having high corrosion resistance in the melt of composition characteristics for molten salts reactors. To date, this essential problem has not been solved [8,9,10]. The experimental data on metallic materials corrosion in molten salts are limited to the corrosion characteristics necessary for the analysis; apart from that, the standards of the experimental conditions are not sufficient or unavailable [11].

Molten salt reactor systems of the IV generation provide efficient fuel usage and cause minimal radioactive waste accumulation at safe and ecologically friendly operation [12]. High temperatures and concentrations of aggressive substances as well as significant rates of the interaction between metallic candidate material and electrolytes are of great concern both from economic and ecological safety perspectives for the molten salt industry [13]. The mechanism of corrosion in molten salts is fundamentally different from that in aqueous media, because the formation of the passive oxide layer on corrosion-resistant alloys becomes thermodynamically impossible [14,15], which limits usage of many known corrosion-resistant alloys [16,17].

Corrosion in the molten salt environment of reactors is associated with several factors including both thermodynamic and kinetic aspects, the formation of fluorides of functional material components as a result of interaction with the medium, and the influence of impurities containing oxygen and water as well as the fission products of spent nuclear fuel (SNF). The majority of functional candidate materials are damaged by selective chromium dissolution in the fluoride formed from the alloy in the electrolyte [18,19,20,21,22]. Moisture and oxides being typical impurities that alongside with the temperature gradient and electrochemical potential difference between various metals are considered to be the corrosion driving forces. Apart from them, doping elements of functional materials dissolved in molten salts boost their corrosion [23].

Currently, nickel-based alloys are considered to be the most promising and, consequently, the most precisely studied corrosion-resistant candidate functional materials. Aluminum is known to be more susceptible to dissolution than other components of the functional materials, and the corrosion of doping components increases from nickel and cobalt to iron, chromium, and aluminum. However, the available literature data are miscellaneous and sometimes contradictory, which is explained by the obvious experimental difficulties including the quality of the salt preparation, purity of the atmosphere over the melt, among others. It is known that plutonium and americium fluorides are imitated by cerium and neodymium fluorides [24,25,26,27,28,29]. It is reasonable first to analyze lanthanide fluorides, as the elements are similar to actinide fluorides’ physical–chemical, thermodynamic, and other properties, and then inquire into the study of individual actinide fluorides and their mixtures. MSR engineering depends greatly on the successful selection and development of the corresponding functional materials. Selection of the alloy with the ideal composition is nearly impossible, because other corrosion-preventive methods are required apart from the material doping.

We have previously studied the corrosion behavior of a number of metallic materials, including iron and nickel austenites of different element and mass compositions of alloys, during short-term corrosion tests. It was found that 12Cr18Ni10Ti and Hastelloy C2000 might be the most appropriate candidate materials for further study as MSR container materials due to the fact of their availability, low cost, and a relatively low corrosion activity in the studied aggressive media. Monel 404 has also demonstrated excellent corrosion resistance in similar temperature conditions [30].

The present paper focused on the study of the corrosion behavior [31,32,33,34,35,36,37,38,39,40,41,42,43,44,45,46,47,48,49] of the FeCrNiTi, NiCrMo, and NiCu alloys during long-term corrosion tests in the high-temperature aggressive alkali fluorides containing cerium trifluoride and neodymium trifluoride as imitators of the nuclear fuel fission products (uranium and plutonium fluorides).

## 2. Materials and Methods

### 2.1. Materials

In this work, the corrosion behavior of steel 12Cr18Ni10Ti steel and Hastelloy C2000 (Ni60Cr20Mo15) and Monel 404 (Ni50Cu50) alloys was investigated. Before testing, the alloys were certified for compliance with the actual composition of the declared one, cut into specimens of a given size convenient for testing, ground, polished successively with abrasive paper of various grain sizes, degreased in an alcohol–acetone mixture, and dried in an oven. The original grade compositions of the materials are presented in Table 1.

**Figure 1:**
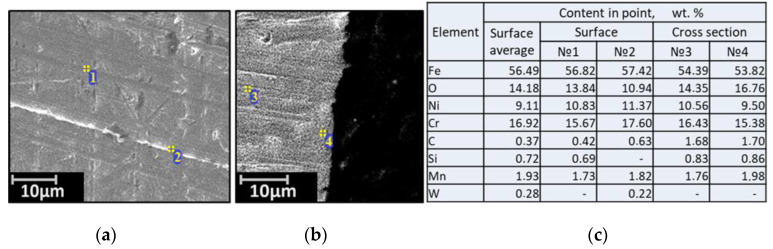
Micrographs of the surface (**a**), the cross-section (**b**), and the elemental composition (**c**) of the source material steel 12Cr18Ni10Ti.

To determine the conformity of the real composition of the studied alloys and their declared composition, metallographic analyses was carried out using a microscope GEOL SM-5900 L. Samples of corrosion-resistant austenitic steel based on iron 12Cr18Ni10Ti were plated rounded on one side in the form of a semicircular segment with a length of 8.56–9.35 mm, width from 6.65 to 6.67, and a thickness from 1.32 to 1.41 mm. The metallographic analyses of the samples of the studied steel is shown in Figure 1.

**Figure 2:**
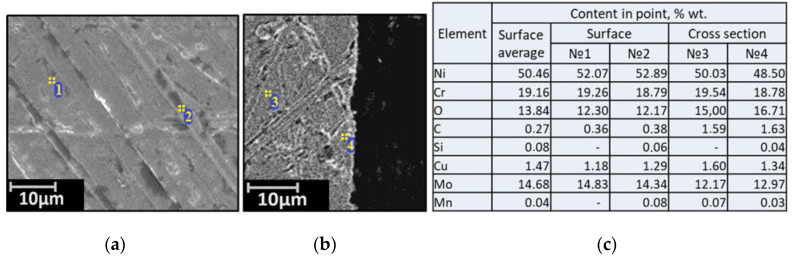
Micrographs of the surface (**a**), the cross-section (**b**), and the elemental composition (**c**) of the source material Hastelloy C2000.

Samples of the Hastelloy C-2000 high nickel alloy consisted of elongated square bars with a length of 6.0–8.13 mm, a width of 2.5–3.08 mm, and a thickness of 2.5–3.0 mm. Metallographic analyses of the Hastelloy C-2000 samples are shown in Figure 2.

**Figure 3:**
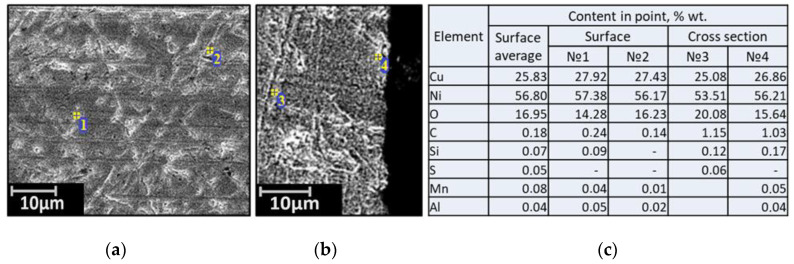
Micrographs of the surface (**a**), the cross-section (**b**), and the elemental composition (**c**) of the source material Monel 404.

Samples of the nickel–copper alloy “Monel 404” were square bars with a length of 6.05–6.12 mm, a width of 6.21–6.24 mm, and a thickness of 5.98 mm. Metallographic analyses of the Monel 404 samples are shown in Figure 3.

According to the results of the metallographic analyses data, the studied metals corresponded to the declared ones.

To prepare the FLiNaK eutectic mixture (46.5 LiF–11.5 NaF–42 KF, mol.%), the following individual salts were used:

− Lithium fluoride LiF of “extra” qualification, mass fraction of LiF: 99.0%;
− Sodium fluoride NaF of analytical grade, mass fraction of NaF: 99.0%;
− Acidic potassium fluoride KHF_2_, analytical grade, mass fraction KHF_2_: 99–101%.

A feature of the developed technique for preparing the FLiNaK eutectic mixture was that instead of the hygroscopic component KF, its acidic anhydrous salt KHF_2_ was used. The melting point of KHF_2_ is 238.7 °C, and the boiling point is in the range 400–500 °C. The decomposition reaction of KHF_2_ takes place already at temperatures of 300–400 °C:

$${\mathrm{KHF}}_{2}\;\to \mathrm{KF}+\mathrm{HF}↑$$

Thus, the introduction of the KHF_2_ component leads not only to the production of anhydrous KF in the FLiNaK eutectic mixture, but also to additional fluorination of impurities. The reactions of formation of volatile compounds of oxygen, sulfur, and phosphorus, which are removed from the melt, can be written as follows:

$$O^{2-}+2\mathrm{HF}\;\to H_{2}O\left(g\right)↑+2F^{-}$$

$$S^{2-}+2\mathrm{HF}\;\to H_{2}S\left(g\right)↑+2F^{-};$$

$$P^{3-}+3\mathrm{HF}\;\to {\mathrm{PH}}_{3}\left(g\right)↑+3F^{-}$$

A crucible from glassy carbon with weighed portions of individual components was placed in a resistance furnace. Heating was carried out at a rate of about 2.5 °C/min to a temperature of 750 °C for 5–6 h. At this temperature, the melt was kept for 2 h. Then, the melt was cooled and transferred to a box with a controlled atmosphere (humidity not more than 2 ppm, oxygen content 2–9 ppm). The prepared melt was stored in a box in a glass container with a tightly closed lid.

### 2.2. Methods

A series of corrosion experiments were performed at 550 °C, the materials were exposed for 100 h in molten alkali fluorides with up to 5 wt.% additions of cerium and neodymium fluorides. All tests were performed in an inert argon glove box atmosphere with the humidity not more than 2 ppm and oxygen concentration not more than 10 ppm.

A specially developed high-temperature experimental bench containing isolated metallic tubes provided physical separation of the working areas of separate parallel experiments, which is required for operation with molten alkali halide melts. Such construction enabled parallel corrosion tests of the samples in the working space of one furnace. In addition, this bench allowed for sampling of the melt during the experiment. The samples of salts’ electrolytes were taken every 8 h to monitor the changes in the composition of corrosion products in the melt.

Five samples of the studied materials were tested in a parallel to increase the data reliability.

To perform a gravimetric analysis, the size of the samples was measured before and after the test. These data were used to calculate the contact area of the materials with the molten salt electrolyte using a digital vernier caliper. The samples were weighted multiple times using an analytical balance MSA 225P (Fisher Scientific, Waltham, MA, USA) with an accuracy of ±0.00001 g.

After the experiments, the samples were rinsed from the remaining fluoride salts in hot boric acid (5 wt.%) solution in the thermostate under continuous stirring at 80 °C for 90 min. Such rinsing fluid has an optimal composition and concentration considering the temperature dependence of the fluoride solubility, which allows for eliminating additional corrosion effects on the materials that were in contact with molten fluorides.

The grade compositions of the studied materials were primarily proved by energy-dispersive X-ray (EDX) microanalysis. The concentration of impurities was characterized using an inductively coupled plasma mass-spectrometer NexIon 2000 (Perkin Elmer, Waltham, MA, USA) and an oxygen/nitrogen/hydrogen elemental analyzer OH836 (Leco Corporation, Joseph, MI, USA).

The changes in the surface morphology and cross-section of the samples were recorded by EDX microanalysis using a JEOL JSM-5900 LV scanning electron microscope (JEOL, Tokyo, Japan).

The solidified melts after the corrosion test, electrolyte probes, and rinsing fluids were subjected to the elemental analysis using a NexION 2000 mass spectrometer (Perkin Elmer, Waltham, MA, USA). When calculating the corrosion rate using gravimetric analysis, the amount of salt remaining in the samples was determined at several points on the sample’s surface using EDX microanalysis, and the value was recalculated for the average surface area to update the real change in the sample’s mass.

## 3. Results and Discussion

### 3.1. Corrosion Rate

Table 2 illustrates the rates of the candidate materials’ corrosion calculated according to gravimetric analysis with the updated real sample mass variation determined by the EDX microanalysis of the unextracted salt in some point for 12Cr18Ni10Ti, Monel 404, and Hastelloy C2000 depending on the electrolyte composition. These samples were preliminary exposed for 100 h in the fluoride melt at 550 °C.

The samples of the 12Cr18Ni10Ti steel demonstrated the largest corrosion rate, on average 1.5 times greater than those of the Hastelloy C2000 alloy and more than five times greater than those of the Monel 404 alloy. As the concentration of cerium and neodymium in fluoride melts increased, the materials’ corrosion rate also increased. Conversely, the addition of cerium fluoride into the melt led to a greater corrosion effect than the addition of neodymium fluoride.

**Figure 4:**
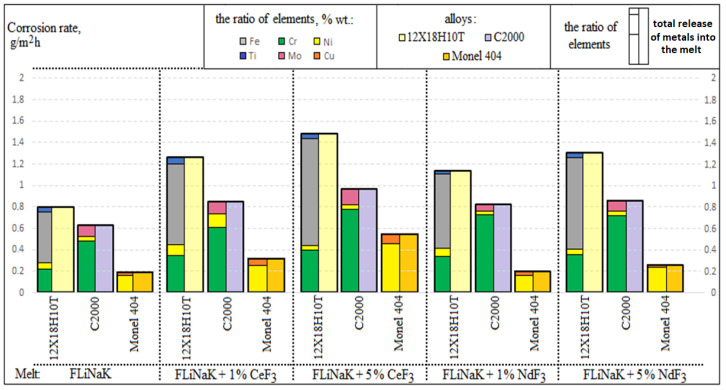
Results of the chemical analysis.

Figure 4 shows the corrosion rates according to the chemical analysis of the melts after the experiments with samples of Monel 404, Hastelloy C2000, and steel 12Cr18Ni10Ti depending on the experimental conditions.

The rates of corrosion, calculated according to the chemical analysis of the molten salts obtained after interacting with the metallic samples, correlated with the values of the corrosion rates obtained by the modified gravimetric analysis. Considering these data, we concluded that the greater the concentration of cerium and neodymium fluorides, the greater the degradation processes in the materials.

A selective dissolution of chromium and titanium was observed in the 12Cr18Ni10Ti steel alongside an active iron transition to the melt. Considering the Hastelloy C2000 alloy, for the most part, chromium transferred to the electrolyte, whereas the nickel concentration was negligible in the frozen probes of the molten salt. The Monel 404 alloy was characterized mainly by the nickel dissolution; the addition of cerium fluoride slightly increased a copper transition to the melt.

**Figure 5:**
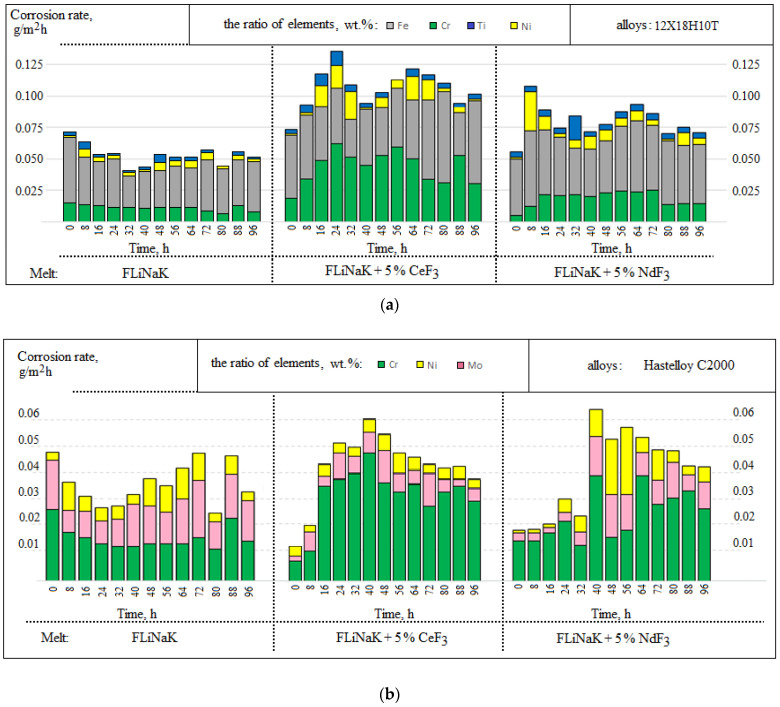

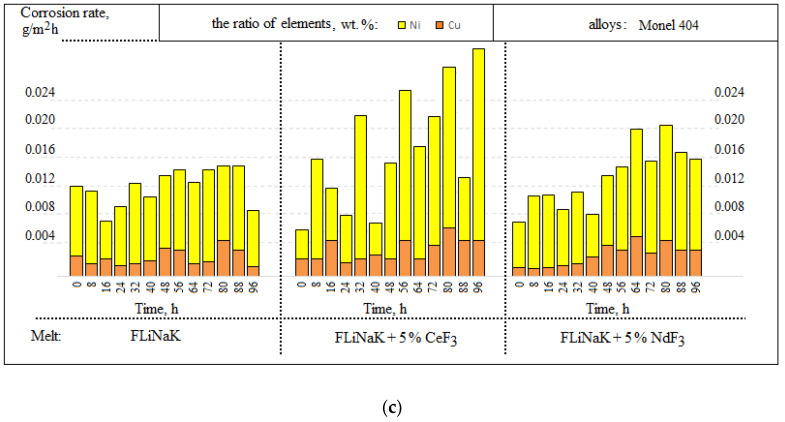
Sampling results of the studied alloys: (**a**) 12Cr18Ni10Ti; (**b**) Hastelloy C2000; (**c**) Monel 404.

Figure 5 illustrates the kinetic characteristics of the transfer of the initial melt components to the salt phase during the corrosion exposure in the alkali metals’ fluorides; the data are presented according to the analysis of the melt samples taken every 8 h.

According to the chemical analysis of the samples, there was a significant increase in the dissolvable Hastelloy C2000, Monel 404, and 12Cr18Ni10Ti components into the electrolyte after 16 h of exposure when cerium and neodymium fluorides were added into the electrolyte. In addition, during the experiments in the FLiNaK melt, a parabolic time dependence of corrosion was recorded. Such behavior may be related to the processes in the subsurface material volume, i.e., the denuding of the inner subsurface layers caused by the dissolution of surface layers during the corrosion tests. Such behavior intermittently increases the total corrosion rate. The corrosion charter was qualified as localized and subsurface.

Apart from that, the Hastelloy C2000 alloy illustrated high selectivity of the molybdenum transfer into the melt, which was not determined by the chemical analysis of the resulting melt (Figure 5b). This may be related with the formation of volatile molybdenum–fluorine compounds of various oxidation degrees at a long-term contact of the material with the corrosive environment during the experiment and their subsequent evacuation from the system. This explains the insignificant deviation in the data of the corrosion rates obtained by the gravimetric and chemical analyses.

### 3.2. SEM of the Studied Samples

The analysis of the corresponding morphological changes in the surfaces of the studied samples was performed by EDX microanalyses of the samples’ surfaces and cross-sections. To clarify the elemental composition of the surface layer, element mapping of the sample cross-section was performed.

**Figure 6:**
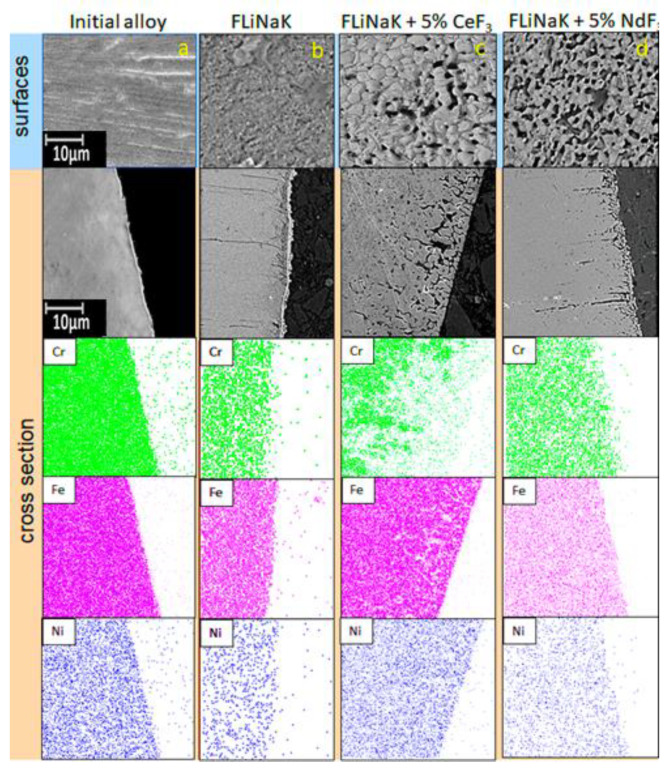
Structure of the surface layers of the 12Cr18Ni10Ti samples after long-term corrosion tests: (**a**) initial; (**b**) exposed in FLiNaK; (**c**) exposed in the FLiNaK + 5 wt.% CeF_3_ melt; (**d**) exposed in the FLiNaK + 5 wt.% NdF_3_ melt.

Figure 6 illustrates the samples of the surfaces and cross-sections for the steel exposed for 100 h in FLiNaK with the addition of cerium and neodymium fluorides at 550 °C.

The EDX microanalyses of the steel samples elucidated that during the corrosion exposure, the pitting corrosion points appeared at the sample’s surface. The addition of cerium fluoride caused the formation of subsurface cavities and significant chromium depletion of the subsurface layer of the alloy bulk. Such cavities may be formed by the development of the initial inter-crystalline corrosion. Addition of neodymium fluoride into the melt led to the formation of relatively deep selective corrosion damage. The ratio between nickel and iron ions remained almost unchanged in the subsurface volume. In other words, additions of cerium and neodymium fluorides into the melt increased the depth of corrosion points, their number, and size. The penetration depths, on average, were 6.2, 18.3, and 12.0 μm for the samples exposed in the FLiNaK, FLiNaK + (5 wt.%) CeF_3_, FLiNaK + (5 wt.%) NdF_3_, respectively. The number of local corrosion defects (per 100 μm^2^) /their size (μm) averages 8.1/0.9, 6.4/4.0, and 7.3/3.1 for samples exposed in FLiNaK, FLiNaK + (5 wt.%) CeF_3_, FLiNaK + (5 wt.%) NdF_3_, respectively.

Apart from that, the surface layer was subjected to large chromium depletion. There were clear signs of the inter-crystalline corrosion at the surface.

**Figure 7:**
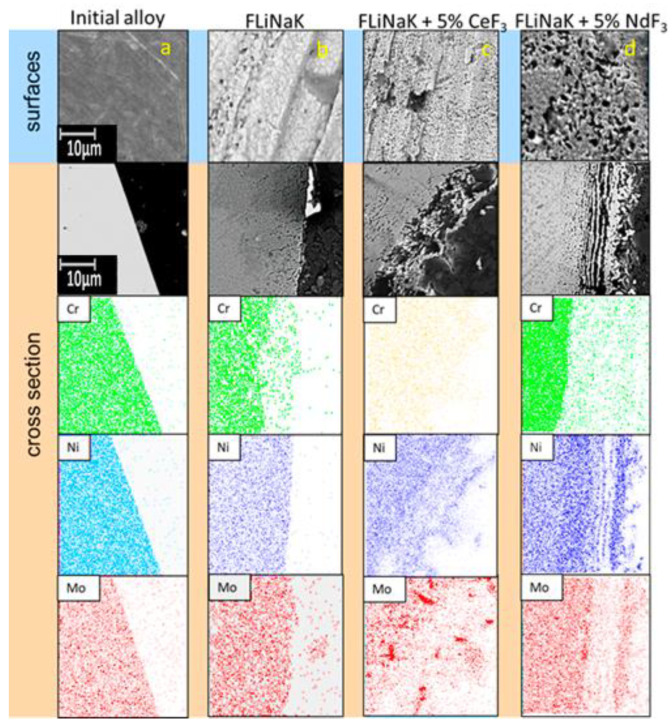
Structure of the surface layers of the samples of Hastelloy C2000 alloy after long-term corrosion tests: (**a**) initial; (**b**) exposed in FLiNaK; (**c**) exposed in the FLiNaK + 5 wt.% CeF_3_ melt; (**d**) exposed in the FLiNaK + 5 wt.% NdF_3_ melt.

Figure 7 presents the SEM images of the surfaces and cross-sections for the Hastelloy C2000 samples that interact with the FLiNaK melt with CeF_3_ or NdF_3_ additions at 550 °C during 100 h.

Multiple localized corrosion points were present at the surface. The redistribution of the molybdenum concentration was observed in the subsurface material volume. The nickel concentration remained almost unchanged. Large chromium depletion was observed at the subsurface layer, which was especially seen at the addition of cerium cations into the melt.

The addition of cerium and neodymium fluorides into the salts’ media increased the candidate material degradation; large-size areas of pitting corrosion were observed alongside with the localized corrosion points as in the case of the samples exposed in lithium, potassium, and sodium fluorides. Apart from that, the element analysis determined that the chromium concentration decreased in the formed cavities and the molybdenum concentration increased near the cavities. On average, the depth of the corrosion was 8.5, 23.2, and 16.9 µm for the samples exposed in FLiNaK, FLiNaK + 5 wt.% CeF_3_, and FLiNaK + 5 wt.% NdF_3_, respectively.

The number of local corrosion defects (per 100 μm^2^)/their size (μm) averaged 6.5/1.1, 18.2/1.2, and 16.7/2.0 for samples exposed in FLiNaK, FLiNaK + (5 wt.%) CeF_3_, and FLiNaK + (5 wt.%) NdF_3_, respectively.

**Figure 8:**
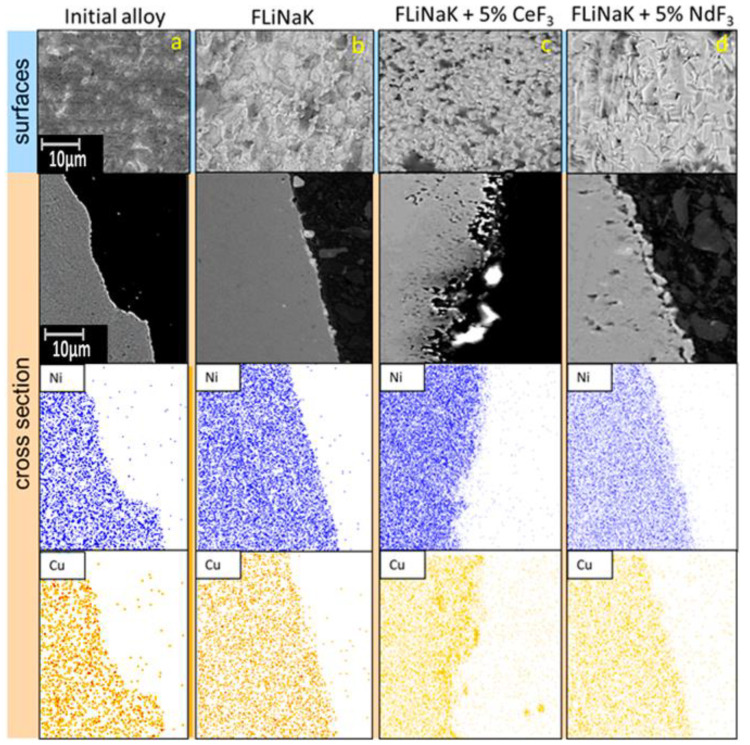
Structure of the surface layers of the Monel 404 samples after long-term corrosion tests: (**a**) initial; (**b**) exposed in FLiNaK; (**c**) exposed in the FLiNaK + 5 wt.% CeF_3_ melt; (**d**) exposed in the FLiNaK + 5 wt.% NdF_3_ melt.

Figure 8 shows the images of the surface and cross-section of the Monel 404 samples exposed in the FLiNaK melt with the addition of cerium or neodymium fluorides.

Some localized corrosion points were observed at the samples’ surfaces. The addition of neodymium fluoride caused etching of the surface layer, which was observed at the surface cross-section; apart from that, the copper concentration in the subsurface layer was found to increase, which testified to the presence of the dissolution of a part of the nickel from the surface. The addition of neodymium fluoride into the melt caused insignificant fragmentary pitting corrosion. It should be noted that insignificant cavities appeared in the subsurface layer at the addition of cerium and neodymium fluorides. Their formation may be associated with the point etching of minor impurities (iron) from the alloy. On average, the corrosion depth was 0.3, 5.2, and 4.0 µm for the samples exposed in FLiNaK, FLiNaK + 5 wt.% CeF_3_, and FLiNaK + 5 wt.% NdF_3_, respectively. The number of local corrosion defects (per 100 μm^2^)/their size (μm) averaged 0/0, 7.3/1.7, and 2.4/0.7 for samples exposed in FLiNaK, FLiNaK + (5 wt.%) CeF_3_, and FLiNaK + (5 wt.%) NdF_3_, respectively.

Thus, it can be concluded that the addition of fluorides of f-elements significantly enhances the local corrosion of materials and increases the size of corrosion foci, their number, and depth.

Considering the data on the Monel 404 alloy, we concluded that the surface morphology was not subjected to any serious changes during the corrosion tests relative to the analogous experiments with the Hastelloy C2000 and 12Cr18Ni10Ti alloys.

We may conclude that Monel 404 demonstrates excellent corrosion resistance in the FLiNaK melt under the studied extremely aggressive conditions. Such a conclusion obtained by the EDX microanalysis is in agreement with the earlier obtained data by the gravimetric and chemical analyses.

However, the actual implementation of the MSR functional materials requires experiments with uranium and plutonium additions into the corrosion media alongside those using imitators of the fission products, i.e., cerium and neodymium.

The corrosion tests with irradiated materials are crucial, because the structure of a great number of materials is known to change under radiation. This may have a significant impact on the anticorrosive properties of the material.

Based on the calculations of the ion force of molten alkali fluorides containing desired concentrations of f-elements fluorides and correlation of the candidate materials according to their corrosion rates, we may conclude that cerium fluoride being a plutonium fluoride imitator creates the highest possible aggressiveness of the electrolyte as opposed to the FLiNaK with the addition NdF_3_. As a result of the performed tests, we may assume that the electrochemical mechanism of the studied corrosion processes has a limiting stage, that is the transition into the chromium and molybdenum salt phase that forms volatile fluorides of various compositions and oxidation degrees under studied temperatures. This causes the formation of subsurface cavities in the samples made of nickel–chromium–molybdenum alloys and pitting corrosion in the samples made of 12Cr18Ni10Ti steel.

## 4. Conclusions

Corrosion resistance of the studied materials was found to decrease in a row from Monel 404 > Hastelloy C2000 > 12Cr18Ni10Ti in a eutectic FLiNaK melt containing cerium fluoride as an imitator of plutonium trifluoride or neodymium fluoride as an imitator of uranium fluoride. Cerium fluoride caused a significantly greater change in the morphology of the candidate materials compared to neodymium fluoride. The addition of fluorides of f-elements significantly enhanced the local corrosion of materials and increased the size of corrosion spots, their number, and depth.

The samples of steel 12Cr18Ni10Ti were subjected to local cracking corrosion. The addition of cerium fluoride into the melt resulted in the additional etching of the alloy surface and deep chromium depletion of the surface. The addition of neodymium fluoride resulted in the formation of the point/inter-crystalline corrosion damage in the sample bulk.

The EDX microanalyses of the Hastelloy C2000 samples illustrated that local corrosion formed at the surface of the samples exposed in the eutectic molten salt electrolyte during the corrosion tests. The addition of cerium and neodymium fluorides resulted in the formation of subsurface vacancies and significant continuous chromium depletion of the subsurface alloy bulk. Moreover, the doping of cerium and neodymium fluorides into the melt increased the fraction of the yield of molybdenum and nickel in the electrolyte.

The samples made of Monel 404 demonstrated an outstanding corrosion resistance under the studied conditions. That is why Monel 404 may be considered a candidate material for molten salt reactors.

## Figures and Tables

**Table 1:** Content of the components in the materials.

Alloy	Elements, wt.%													
	Fe	C	Si	Mn	Ni	S	P	Cr	Ti	W	Cu	Co	Al	Mo
Monel 404	<0.5	<0.15	<0.1	<0.1	52.0–57.0	<0.024	-	-	-	-	Base	-	<0.05	-
Hastelloy C2000	<3.0	<0.01	<0.08	<0.05	Base	<0.01	<0.025	22.0–24.0	-	-	1.3–1.9	<2.0	<0.5	15.0–17.0
Steel 12Cr18Ni10Ti	Base	<0.08	<0.8	<2.0	9.0–11.0	<0.02	<0.035	17.0–19.0	0.5–0.8	-	<0.1	-	-	-

**Table 2:** Corrosion rate of the studied materials with an experiment time of 100 h.

Studied Fluoride Melt	Corrosion Rate, g/m^2^h		
	Alloy		
	12Cr18Ni10Ti	Hastelloy C2000	Monel 404
FLiNaK	0.806 ± 0.040	0.597 ± 0.030	0.122 ± 0.006
FLiNaK + 1 wt.% CeF_3_	1.260 ± 0.063	0.857 ± 0.043	0.222 ± 0.011
FLiNaK + 5 wt.% CeF_3_	1.483 ± 0.074	0.948 ± 0.047	0.294 ± 0.015
FLiNaK + 1 wt.% NdF_3_	1.138 ± 0.057	0.805 ± 0.040	0.137 ± 0.007
FLiNaK + 5 wt.% NdF_3_	1.303 ± 0.065	0.854 ± 0.043	0.167 ± 0.008
